# Deferred Versus Immediate Stenting in Late-Presenting ST-Segment Elevation Myocardial Infarction (STEMI) Patients With a High Thrombus Burden: A Retrospective Analysis

**DOI:** 10.7759/cureus.86317

**Published:** 2025-06-18

**Authors:** Ahmed Hesham Hammad, Mahmoud Abdelaziz Ismaiel, Mohammed H. Abd-Elnaby, Attaa Khaleel Taha, Hamza Kabil

**Affiliations:** 1 Cardiology, Faculty of Medicine, Mansoura University, Mansoura, EGY; 2 Cardiology, Global Medical City Hospital, Cairo, EGY; 3 Cardiology, College of Medicine, University of Baghdad, Baghdad, IRQ; 4 Cardiology, Faculty of Medicine, Damietta University, Mansoura, EGY

**Keywords:** high thrombus burden, late presentation, major adverse cardiac events (mace), primary percutaneous coronary intervention (pci), st-elevation myocardial infarction (stemi)

## Abstract

Background: Late-presenting patients with ST-segment elevation myocardial infarction (STEMI), defined as those presenting ≥12 hours after symptom onset, represent a high-risk and often underrepresented population. Despite evidence supporting primary percutaneous coronary intervention (PCI) in these patients, those with a high thrombus burden (HTB) face an increased risk of no reflow and adverse outcomes, particularly with immediate stenting. The optimal stent timing in this subgroup remains unclear.

Methods: This retrospective study included 200 patients with late-presenting STEMI and angiographically confirmed HTB. Patients were assigned to either the deferred stenting (DS; n=100) group or the immediate stenting (IS; n=100) group. Clinical and procedural outcomes, including myocardial blush grade (MBG), thrombolysis in myocardial infarction (TIMI) flow, and major adverse cardiovascular events (MACE) at one year, were compared.

Results: The DS group was associated with significantly lower no reflow (11 (11%) vs. 26 (26%), p=0.01), less distal embolization (6 (6%) vs. 16 (16%), p=0.04), and higher post-PCI TIMI grade 3 flow (73 (73%) vs. 57 (57%), p=0.03) and MBG 3 (58 (58%) vs. 36 (36%), p=0.01). One-year MACE was significantly reduced in the DS group (7 (7%) vs. 20 (20%), p=0.01). The DS group was independently associated with improved myocardial perfusion and lower MACE.

Conclusion: In late-presenting STEMI patients with HTB, deferred stenting yielded superior perfusion and clinical outcomes compared to immediate stenting. These findings underscore the importance of individualized stent timing strategies in this high-risk, often overlooked subgroup.

## Introduction

Primary percutaneous coronary intervention (PCI) with flow restoration and stent implantation in the infarct-related artery (IRA) is the cornerstone treatment for ST-segment elevation myocardial infarction (STEMI) [1]. However, no reflow is a relatively common complication during PCI. This is primarily attributed to distal embolization, resulting in microvascular obstruction. It often manifests as reduced thrombolysis in myocardial infarction (TIMI) flow in the culprit artery post-stenting [2], culminating in poor myocardial perfusion despite good epicardial flow [3]. Preventing no reflow remains a significant challenge. Multiple studies have failed to confirm the efficacy of distal protection devices in preventing no reflow [4], while thrombectomy shows potential benefit in selected cases, particularly those with a high thrombus burden (HTB) [5,6].

HTB complicates PCI and renders the optimal timing for stent placement controversial. Immediate stenting in patients with HTB is associated with a higher risk of no reflow [7,8]. Once flow is restored, a deferred stenting strategy may be considered. Several studies have demonstrated that deferring stent implantation in HTB cases improves procedural outcomes and leads to better short- and long-term results, including improved ejection fraction and reduced major adverse cardiovascular events (MACE) [9,10]. The Impact of Immediate Stent Implantation Versus Deferred Stent Implantation on Infarct Size and Microvascular Perfusion in Patients With ST-Segment-Elevation Myocardial Infarction (INNOVATION) trial highlighted the potential benefit of deferred stenting in reducing infarct size and microvascular obstruction (MVO) [11]. However, more recent randomized controlled trials like the Third Danish Study of Optimal Acute Treatment of Patients With ST-Elevation Myocardial Infarction-Deferred Stenting (DANAMI-3 DEFER) and the Minimalist Immediate Mechanical Intervention (MIMI) trial have shown inconsistent results regarding routine stent deferral, and no specific subgroup analyses were conducted [12,13].

Late-presenting STEMI patients (12-48 hours after symptom onset) generally have larger infarcts, worse outcomes, and higher complication rates than early presenters (<12 hours) [14]. Nonetheless, PCI still achieves considerable myocardial salvage and improved outcomes compared to medical therapy alone in this subset [14,15]. While the impact of stent timing in HTB cases has been studied, there is limited evidence specific to late presenters. This study aimed to address this gap by evaluating whether deferred stenting further improves outcomes in this patient group.

## Materials and methods

Study design

This retrospective study was conducted at the Cardiology Department of Mansoura University Hospital, Egypt. It included 200 adult patients (aged >18 years) of both sexes, all diagnosed with STEMI, presenting ≥12 hours after symptom onset, and confirmed to have HTB by angiography. Patients were enrolled between 2023 and 2024.

Inclusion criteria were late presentation (≥12 hours) with ongoing symptoms, thrombus burden score (TBS) ≥3 in the IRA, and successful re-establishment of TIMI grade 2-3 flow before deciding on stenting. Exclusion criteria included cardiogenic shock persisting after supportive therapy despite successful reperfusion at the time the stent-timing decision was made, IRA involving unprotected left main coronary artery or saphenous vein grafts, and failure to achieve TIMI grade 2-3 flow before stenting [16].

Participants were divided into two groups: the deferred stenting (DS; n=100) group, which underwent stenting 24-48 hours after initial recanalization with enhanced antithrombotic therapy, and the immediate stenting (IS; n=100) group, which received stents during the initial PCI.

The study received approval from the Medical Research Ethics Committee of the Faculty of Medicine, Mansoura University, and was conducted in accordance with the Declaration of Helsinki (2013) [17].

Study tools

Data were collected retrospectively from medical records and included demographic details, comorbidities, cardiovascular risk factors (e.g., diabetes, hypertension, smoking), prior cardiac history, and family history of coronary artery disease (CAD).

Clinical data on initial presentation included ischemic time, baseline troponin levels, Killip-Kimball heart-failure classification (Killip class), and left ventricular ejection fraction (LVEF). PCI details were recorded, including need for manual thrombectomy, use of glycoprotein IIb/IIIa inhibitors, contrast volume, stent specifications, and intraprocedural complications.

The IRA was identified using standard 12-lead electrocardiogram (ECG) findings, baseline echocardiography, and angiographic features. Thrombus burden was classified on a scale from grade 0 to 5 as absent, low, moderate, or high [16]. Myocardial blush grade (MBG) was assessed according to van’t Hof et al.’s scale (grades 0-3) [18]. Multivessel disease was defined as ≥50% stenosis in ≥1 major epicardial artery distinct from the IRA.

Distal embolization was defined as a contrast filling defect with abrupt cutoff distal to the angioplasty site. No reflow was defined as TIMI grade 0-1 flow in the absence of occlusion or visible distal embolization. MACE (cardiac death, reinfarction, heart failure, target vessel revascularization (TVR)) was evaluated at one month and one year.

Statistical analysis

Data were analyzed using IBM SPSS Statistics for Windows, Version 23.0 (IBM Corp., Armonk, USA). Quantitative variables were expressed as mean ± standard deviation (SD) and qualitative variables as frequencies or percentages. Normality was tested using the Shapiro-Wilk test. Chi-square or Fisher’s exact test was used for categorical variables. An independent sample t-test was used for continuous variables between the two groups. Multivariable logistic regression (forward method) was applied to identify predictors of inadequate reperfusion (MBG ≤1), including factors with p<0.05 in univariable analysis. Kaplan-Meier analysis was used for time-to-event data, with comparisons via the log-rank test. A p-value ≤0.05 was considered statistically significant.

## Results

This study included 200 patients diagnosed with late-presenting STEMI and HTB, divided equally into the DS group (n=100) and the IS group (n=100). Patients in the DS group underwent stent implantation ≥24 hours after initial recanalization with intensified antithrombotic therapy, while those in the IS group received stents during the initial PCI.

Baseline characteristics of the two groups are summarized in Table 1. The age of the participants ranged from 40 to 79 years, with a mean ± SD of 59.0 ± 11.2 years in the DS group and 57.5 ± 11.8 years in the IS group. The DS group included 53 (53%) male and 47 (47%) female patients, while the IS group included 42 (42%) male and 58 (58%) female patients. Baseline characteristics showed no significant differences between the two groups.

**Table 1:** Baseline characteristics of the study groups Abbreviations: CAD, coronary artery disease; DM, diabetes mellitus; DS, deferred stenting; FH, family history; HTN, hypertension; IS, immediate stenting; LVEF, left-ventricular ejection fraction; SD, standard deviation. Statistical tests: t = independent sample t-test; χ² = Pearson chi-square test; Fisher = Fisher’s exact test. Non-significant if p>0.05 and significant if p<0.05.

Variables		DS group (n=100)	IS group (n=100)	Test	p-value
Age (years)	Mean ± SD	59 ± 11.2	57.5 ± 11.8	t = 0.941	0.35
	Range	(40-79)	(40-79)		
Sex (n, %)	Male	53 (53%)	42 (42%)	χ² = 2.43	0.12
	Female	47 (47%)	58 (58%)		
Risk factors (n, %)	HTN	49 (49%)	41 (41%)	χ² = 1.29	0.26
	DM	40 (40%)	29 (29%)	χ² = 2.68	0.1
	Dyslipidemia	52 (52%)	44 (44%)	χ² = 1.28	0.26
	Smoker	33 (33%)	27 (27%)	χ² = 0.858	0.65
	FH of CAD	8 (8%)	15 (15%)	Fisher	0.18
Ischemic time (hours)	Mean ± SD	26.7 ± 8.13	28 ± 8.41	t = 1.103	0.27
	Range	(12-39)	(12-39)		
Initial troponin (ng/ml)	Mean ± SD	12.8 ± 8.08	12.1 ± 7.59	t = 0.656	0.51
	Range	(2.5-35)	(2.5-35)		
Killip class on admission (n, %)	Ⅰ	36 (36%)	43 (43%)	Fisher	0.26
	Ⅱ	23 (23%)	29 (29%)		
	Ⅲ	26 (26%)	16 (16%)		
	Ⅳ	15 (15%)	12 (12%)		
Baseline LVEF (%)	Mean ± SD	40.9 ± 7.2	42.4 ± 7.42	t = 1.46	0.15
	Range	(30-54)	(30-55)		

Table 2 presents the angiographic characteristics of the DS and IS groups. All characteristics were comparable between the two groups (all p >0.05). Table 3 gives procedural details, which differed significantly between the two groups. Manual thrombectomy was more frequently performed in the IS group compared to the DS group (79 (79%) vs. 63 (63%), p=0.01). No reflow occurred in 26 (26%) participants of the IS group and 11 (11%) participants of the DS group (p=0.01). Distal embolization was also higher in the IS group (16 (16%)) compared to the DS group (16 (16%) vs. 5 (5%), p=0.04).

**Table 2:** Angiographic characteristics of the study groups Abbreviations: DS, deferred stenting; IS, immediate stenting; LAD, left anterior descending artery; LCX, left circumflex artery; MBG, myocardial blush grade; RCA, right coronary artery; TIMI, thrombolysis in myocardial infarction; TBS, thrombus burden score; SD, standard deviation. Statistical tests: t = independent sample t-test; χ² = Pearson chi-square test; Fisher = Fisher’s exact test. Non-significant if p>0.05 and significant if p<0.05.

Variables		DS group (n=100)	IS group (n=100)	Test	p-value
Culprit vessel (n, %)	LAD	35 (35%)	34 (34%)	χ² = 0.633	0.73
	LCX	32 (32%)	28 (28%)		
	RCA	33 (33%)	38 (38%)		
Vessel diameter (mm)	Mean ± SD	3.04 ± 0.62	2.99 ± 0.57	t = 0.530	0.59
	Range	(2.01-3.99)	(2.00-3.98)		
Initial TIMI flow grade (n, %)	0	40 (40%)	42 (42%)	Fisher	0.9
	1	30 (30%)	32 (32%)		
	2	18 (18%)	17 (17%)		
	3	12 (12%)	9 (9%)		
Initial TBS	Mean ± SD	4.03 ± 0.87	3.98 ± 0.82	t = 0.419	0.68
	Range	(3-5)	(3-5)		
Initial MBG	0	55 (55%)	56 (56%)	Fisher	0.69
	1	31 (31%)	25 (25%)		
	2	11 (11%)	14 (14%)		
	3	3 (3%)	5 (5%)		

**Table 3:** Procedure details of the study groups Abbreviations: DS, deferred stenting; GP Ⅱb/Ⅲa inhibitor, glycoprotein Ⅱb/Ⅲa platelet inhibitor; IS, immediate stenting; PCI, percutaneous coronary intervention; SD, standard deviation. Statistical tests: t = independent sample t-test; χ² = Pearson chi-square test; Fisher = Fisher’s exact test. Non-significant if p>0.05 and significant if p<0.05.

Variables		DS group (n=100)	IS group (n=100)	Test	p-value
Manual thrombectomy (n, %)	No	37 (37%)	21 (21%)	χ² = 6.22	0.01
	Yes	63 (63%)	79 (79%)		
GP Ⅱb/Ⅲa inhibitor use (n, %)	No	11 (11%)	16 (16%)	Fisher	0.41
	Yes	89 (89%)	84 (84%)		
Contrast volume (ml)	Mean ± SD	181 ± 73.4	177 ± 71.6	t = 0.370	0.71
	Range	(51-298)	(52-299)		
Complications during PCI (n, %)	No reflow	11 (11%)	26 (26%)	Fisher	0.01
	Coronary perforation	2 (2%)	3 (3%)	Fisher	1.00
	Distal embolization	6 (6%)	16 (16%)	Fisher	0.04
Stent length (mm)	Mean ± SD	26.7 ± 8.57	27.3 ± 7.88	t = 0.472	0.64
	Range	(11-42)	(12-41)		
Stent diameter (mm)	Mean ± SD	3.24 ± 0.46	3.22 ± 0.44	t = 0.314	0.75
	Range	(2.5-4)	(2.5-4)		

Table 4 summarizes the interventional and clinical outcomes of the two groups. Post-PCI TIMI grade 3 flow was achieved in 73 (73%) cases of the DS group and 57 (57%) cases of the IS group (p=0.03). MBG 3 was observed in 58 (58%) participants of the DS group compared to 36 (36%) participants of the IS group (p=0.01). Complete thrombus resolution occurred more frequently in the DS group in comparison to the IS group (83 (83%) vs. 67 (67%), p=0.01). Although the 30-day MACE rate was similar, the one-year cumulative MACE rate was significantly lower in the DS group when compared to the IS group (7 (7%) vs. 20 (20%), p=0.01). Reinfarction occurred exclusively in the IS group (p=0.03). There was no significant difference in mortality, heart failure incidence, or TVR. Hospital stay was significantly longer in the IS group (p=0.03). Follow-up LVEF was higher in the DS group (p=0.02), with greater LVEF improvement observed in DS group patients (p<0.001).

**Table 4:** Outcomes of the study groups Abbreviations: DS, deferred stenting; IS, immediate stenting; LVEF, left-ventricular ejection fraction; MACE, major adverse cardiac events; MBG, myocardial blush grade; PCI, percutaneous coronary intervention; TIMI, thrombolysis in myocardial infarction; TVR, target vessel revascularization; SD, standard deviation. Statistical tests: t = independent sample t-test; χ² = Pearson chi-square test; Fisher = Fisher’s exact test. Non-significant if p>0.05 and significant if p<0.05.

Variables		DS group (n=100)	IS group (n=100)	Test	p-value
Post-PCI TIMI flow grade (n, %)	0	3 (3%)	10 (10%)	Fisher	0.03
	1	6 (6%)	14 (14%)		
	2	18 (18%)	19 (19%)		
	3	73 (73%)	57 (57%)		
Post-PCI MBG (n, %)	0	8 (8%)	16 (16%)	Fisher	0.01
	1	12 (12%)	20 (20%)		
	2	22 (22%)	28 (28%)		
	3	58 (58%)	36 (36%)		
Thrombus resolution (n, %)	No	17 (17%)	33 (33%)	Fisher	0.01
	Yes	83 (83%)	67 (67%)		
30-day MACE (n, %)	Total	2 (2%)	3 (3%)	Fisher	1.00
	Cardiac death	1 (1%)	1 (1%)	Fisher	1.00
	Reinfarction	0 (0%)	1 (1%)	Fisher	1.00
	Heart failure	1 (1%)	1 (1%)	Fisher	1.00
One-year MACE (n, %)	Total	7 (7%)	20 (20%)	Fisher	0.01
	Cardiac death	4 (4%)	7 (7%)	Fisher	0.33
	Reinfarction	0 (0%)	6 (6%)	Fisher	0.03
	Heart failure	3 (3%)	4 (4%)	Fisher	1.00
	TVR	0 (0%)	3 (3%)	Fisher	0.25
Hospital stay (days)	Mean ± SD	6.89 ± 2.58	7.79 ± 3.13	t = 2.219	0.03
	Range	(2-10)	(3-13)		
Follow-up LVEF (%)	Mean ± SD	48.3 ± 7.29	45.9 ± 6.96	t = 2.340	0.02
	Range	(36-60)	(33-60)		
LVEF improvement (ΔEF)	Mean ± SD	6.75 ± 4.6	3.53 ± 3.15	t = 5.77	<0.001
	Range	(1-17)	(1-16)		

Table 5 presents the multivariable logistic regression model of predictors of poor myocardial perfusion (MBG≤1). Immediate stenting (odds ratio (OR)=2.84, 95% confidence interval (CI): 1.31-6.14, p=0.008) and high baseline TBS (OR=2.09, 95% CI: 1.31-3.33, p=0.002) were identified as independent predictors of poor myocardial perfusion (MBG≤1).

**Table 5:** Logistic regression analysis of predictors of inadequate myocardial reperfusion (MBG≤1) Abbreviations: CI, confidence interval; DM, diabetes mellitus; HTN, hypertension; LVEF, left-ventricular ejection fraction; MACE, major adverse cardiac events; MBG, myocardial blush grade; TBS, thrombus burden score; TIMI, thrombolysis in myocardial infarction.

Variables	Univariate analysis		Multivariate analysis	
	p-value	Odds (CI 95%)	p-value	Odds (CI 95%)
Age	0.65	0.99 (0.96-1.02)	-	-
Sex	0.21	1.58 (0.77-3.24)	-	-
HTN	0.81	1.09 (0.53-2.24)	-	-
DM	0.29	1.53 (0.69-3.38)	-	-
Dyslipidemia	0.78	1.11 (0.54-2.26)	-	-
Smoking	0.33	0.67 (0.29-1.51)	-	-
Ischemic time	0.03	1.04 (1.17-1.93)	0.41	1.01 (0.22-1.82)
Vessel diameter	0.64	1.16 (0.63-2.11)	-	-
Initial TIMI	0.04	0.76 (0.39-0.85)	0.19	0.79 (0.91-1.17)
Initial TBS	<0.001	2.19 (1.47-3.27)	0.002	2.09 (1.31-3.33)
Baseline LVEF	0.01	0.55 (0.48-0.63)	0.24	0.93 (0.83-1.04)
Hospital stay	0.83	1.01 (0.89-1.15)	-	-
Immediate stent	<0.001	2.91 (1.54-8.23)	0.008	2.84 (1.31-6.14)

Table 6 displays the Cox proportional hazards model for predictors of one-year MACE. Cox regression analysis showed that immediate stenting (hazard ratio (HR)=1.17, p<0.001), ischemic time (HR=1.54, p=0.05), initial TIMI flow (HR=0.07, p=0.007), TBS (HR=1.95, p=0.006), and no reflow (HR=2.31, p=0.001) were significantly associated with increased one-year MACE risk.

**Table 6:** Multivariate Cox regression analysis of predictors of one-year MACE Abbreviations: CI, confidence interval; HR, hazard ratio; MACE, major adverse cardiac events; TIMI, thrombolysis in myocardial infarction; TBS, thrombus burden score; MBG, myocardial blush grade; HTN, hypertension; DM, diabetes mellitus.

Variables	One-year MACE		
	HR	95% CI	p-value
Age	0.98	(0.95-1.02)	0.36
Sex	1.13	(0.53-2.42)	0.75
DM	1.41	(0.91-2.23)	0.13
HTN	1.05	(0.49-2.26)	0.89
Dyslipidemia	1.41	(0.66-3.01)	0.38
Smoking	0.97	(0.39-2.42)	0.94
Immediate stenting	1.17	(1.85-2.82)	<0.001
Initial MBG	0.42	(0.54-3.73)	0.48
Ischemic time	1.54	(1.02-2.33)	0.05
Initial TIMI flow	0.07	(0.01-0.48)	0.007
TBS	1.95	(1.23-3.83)	0.006
No reflow phenomenon	2.31	(1.42-3.87)	0.001

**Figure 1:**
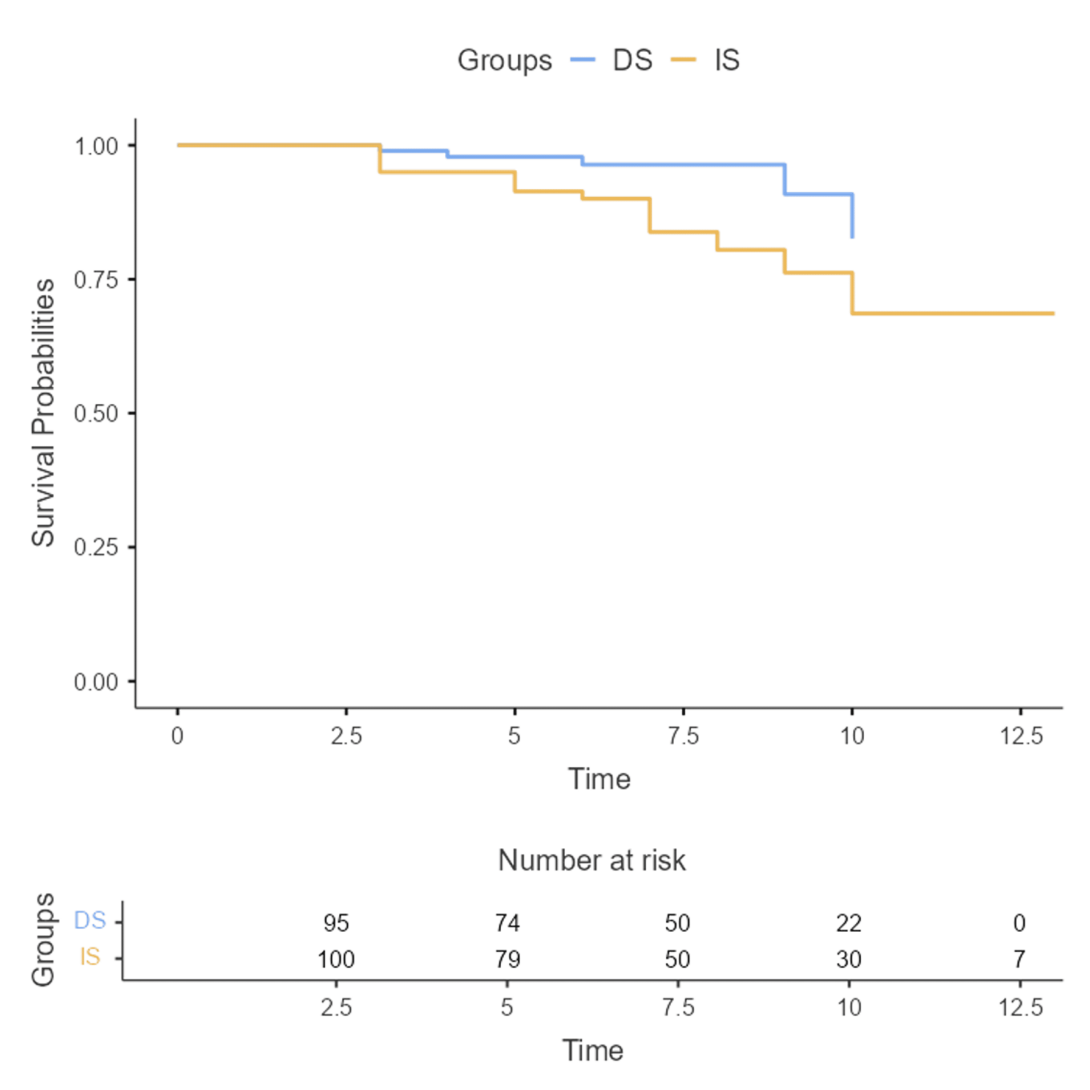
Kaplan-Meier curve of survival free from MACE comparing the DS and IS groups Abbreviations: MACE, major adverse cardiac events; DS, deferred stenting; IS, immediate stenting

Figure 1 shows the Kaplan-Meier analysis demonstrating a significantly lower cumulative incidence of MACE in the DS group compared to the IS group (log-rank p=0.01).

## Discussion

Primary PCI of the IRA remains the first-line intervention to restore perfusion and reduce infarct size of STEMI. Stents are widely used to stabilize plaques and prevent acute occlusion or restenosis [1,19]. In patients with HTB, deferred stenting has gained support as an alternative to immediate stenting. Delaying stent deployment allows for thrombus reduction through pharmacologic therapy, potentially lowering the risk of complications such as no reflow, as supported by meta-analyses [20,21]. 

This study compared deferred versus immediate stenting in 200 late-presenting STEMI patients (DS group: n=100; IS group: n=100). No significant differences were found in baseline characteristics. There was a statistically significant difference in clinical outcomes between the two groups, with post-PCI TIMI flow grade 3 achieved in 73 (73%) of DS group patients compared to 57 (57%) of IS group patients (p=0.03). This aligns with Magdy et al., who found no/slow reflow significantly higher in the IS group and a higher final TIMI flow grade 3 in the DS group (82.2% vs. 76%) [22]. Similarly, Ghazal et al. showed TIMI grade 3 flow in 81% of DS patients compared to 63% of IS patients (p=0.03) [23]. Liu et al. reported higher TIMI grade 3 flow in the DS group (97.6%) versus the IS group (86.8%) (p<0.05) [24]. Tang et al. found significant thrombus burden reduction after seven days of deferral with higher final TIMI grade 3 flow [25]. However, Belle et al. reported no significant difference in TIMI grade 3 flow between IS (94%) and DS (96.7%) groups [13].

A potential mechanical explanation for the improvement associated with delayed stenting may actually be the reduction in thrombus burden. In all the studies where quantitative coronary analysis was performed, a significant reduction in thrombus burden was observed before and after the interval required for delayed stenting [25].

Our results showed that distal embolization was observed in 16 (16%) patients in the IS group compared to six (6%) patients in the DS group (p=0.04). Similar results were reported by Ghazal et al., as in their study, distal embolization occurred less frequently in the DS group (7% vs. 11.1%) (p=0.1) [23].

In this study, post-PCI MBG 3 was observed in 58 (58%) of the DS group patients compared to 36 (36%) patients of the IS group (p=0.01). This aligns with Ghazal et al., who showed MBG 3 in 29 patients of the IS group (53%) versus 39 patients of the DS group (72%) with a significant difference (p=0.04) [23]. Liu et al. reported MBG 3 was significantly lower in the IS group (84.5%) versus the DS group (97.6%) (p<0.05) [24]. Tang et al. also found MBG 3 at PCI end was 73.9% in the IS group versus 94.9% in the DS group (p=0.017), with MBG 0-1 more frequent in IS (19.6% vs. 0%, p=0.003) [25].

In the current study, complete thrombus resolution was more frequent in the DS group compared to the IS group (83 (83%) vs. 67 (67%), p=0.01). This aligns with Carrick et al. in the Randomized Trial of Deferred Stenting Versus Immediate Stenting to Prevent No- or Slow-Reflow in Acute ST-Segment Elevation Myocardial Infarction (DEFER-STEMI), who showed significantly lower angiographic thrombus at the second procedure compared to the first (62.7% vs. 98.1%, p<0.0001) [10].

The current findings showed no significant difference between the two groups regarding stent length and diameter. This aligns with Ghazal et al., who reported mean stent diameters of 3.2±0.37 mm and 3.3±0.39 mm and lengths of 29±7.4 mm and 27±6.9 mm in IS and DS groups, respectively, with no significant difference (p=0.16 and 0.1) [23]. Liu et al. also found similar stent diameters (3.10±0.47 mm vs. 3.12±0.42 mm, p=0.759) and lengths (21.61±5.15 mm vs. 18.00±4.30 mm, p=0.00) between the two groups [24]. This agrees with the DEFER-STEMI trial, which showed reduced lesion length after stent deferral but no significant difference between IS and DS groups, likely due to spontaneous and pharmacologic vessel changes over time resulting in shorter stents with better long-term prognosis and lower restenosis rates [10].

In our results, follow-up LVEF was significantly higher in the DS group (p=0.02), with more pronounced improvement among DS group patients (p<0.001). Ghazal et al. reported similar significant differences after six months, with higher LVEF in the DS group than in the IS group (49.67±5.75% vs. 45.57±6.53%, p<0.05) and differences in stroke volume index (SVI) [23]. Jolicoeur et al. (2020) also showed greater LVEF improvement in the DS group [26], while Kelbæk et al. (2016) found higher LVEF in deferred stent implantation patients than in conventional PCI (p=0.043) [12].

In the current study, although the 30-day MACE rate was not significantly different, the one-year cumulative MACE rate was significantly lower in the DS group compared to the IS group (7 (7%) vs. 20 (20%), p=0.01). This aligns with Magdy et al., who found no significant difference in in-hospital overall MACE, bleeding, and contrast-induced nephropathy (CIN) between DS and IS groups, but at six months, immediate stenting showed a higher risk of MACE compared to 4-16 hour and seven day deferral groups (p=0.034 and p=0.038, respectively) [22]. Tang et al. reported no MACE during hospitalization in both groups, with no significant difference at six months, but lower heart failure incidence in the deferral group (5.0% vs. 19.1%) [25]. Kelbæk et al. (2016) showed no significant difference in MACE (all-cause mortality, rehospitalization for heart failure, non-fatal myocardial infarction, TVR) between IS and DS groups over 42 months (p>0.05) [12]. Kelbæk et al. found similar composite primary endpoint rates (18% vs. 17%) in conventional PCI and DS groups [12].

## Conclusions

Both deferred and immediate stenting strategies are effective in managing late-presenting STEMI patients undergoing PCI. However, deferred stenting was associated with superior outcomes, including lower rates of no reflow and distal embolization, improved myocardial perfusion (TIMI grade 3 flow and MBG 3), more complete thrombus resolution, better LVEF improvement, shorter hospital stays, and significantly reduced one-year MACE rates. These findings support considering a deferred stenting approach in patients with HTB presenting beyond 12 hours after symptom onset.

## References

[1] Neumann FJ, Sousa-Uva M, Ahlsson A (2019). 2018 ESC/EACTS guidelines on myocardial revascularization. Eur Heart J.

[2] Konijnenberg LS, Damman P, Duncker DJ (2020). Pathophysiology and diagnosis of coronary microvascular dysfunction in ST-elevation myocardial infarction. Cardiovasc Res.

[3] Harrison RW, Aggarwal A, Ou FS, Klein LW, Rumsfeld JS, Roe MT, Wang TY (2013). Incidence and outcomes of no-reflow phenomenon during percutaneous coronary intervention among patients with acute myocardial infarction. Am J Cardiol.

[4] Stone GW, Webb J, Cox DA (2005). Distal microcirculatory protection during percutaneous coronary intervention in acute ST-segment elevation myocardial infarction: a randomized controlled trial. JAMA.

[5] Vlaar PJ, Svilaas T, van der Horst IC (2008). Cardiac death and reinfarction after 1 year in the thrombus aspiration during percutaneous coronary intervention in acute myocardial infarction study (TAPAS): a 1-year follow-up study. Lancet.

[6] Kaltoft A, Bøttcher M, Nielsen SS (2006). Routine thrombectomy in percutaneous coronary intervention for acute ST-segment-elevation myocardial infarction: a randomized, controlled trial. Circulation.

[7] Sianos G, Papafaklis MI, Daemen J (2007). Angiographic stent thrombosis after routine use of drug-eluting stents in ST-segment elevation myocardial infarction: the importance of thrombus burden. J Am Coll Cardiol.

[8] Henriques JP, Zijlstra F, Ottervanger JP, de Boer MJ, van't Hof AW, Hoorntje JC, Suryapranata H (2002). Incidence and clinical significance of distal embolization during primary angioplasty for acute myocardial infarction. Eur Heart J.

[9] Ke D, Zhong W, Fan L, Chen L (2012). Delayed versus immediate stenting for the treatment of ST-elevation acute myocardial infarction with a high thrombus burden. Coron Artery Dis.

[10] Carrick D, Oldroyd KG, McEntegart M (2014). A randomized trial of deferred stenting versus immediate stenting to prevent no- or slow-reflow in acute ST-segment elevation myocardial infarction (DEFER-STEMI). J Am Coll Cardiol.

[11] Kim JS, Lee HJ, Woong Yu C (2016). INNOVATION study (impact of immediate stent implantation versus deferred stent implantation on infarct size and microvascular perfusion in patients with ST-segment-elevation myocardial infarction). Circ Cardiovasc Interv.

[12] Kelbæk H, Høfsten DE, Køber L (2016). Deferred versus conventional stent implantation in patients with ST-segment elevation myocardial infarction (DANAMI 3-DEFER): an open-label, randomised controlled trial. Lancet.

[13] Belle L, Motreff P, Mangin L (2016). Comparison of immediate with delayed stenting using the minimalist immediate mechanical intervention approach in acute ST-segment-elevation myocardial infarction: the MIMI study. Circ Cardiovasc Interv.

[14] McNair PW, Bilchick KC, Keeley EC (2019). Very late presentation in ST elevation myocardial infarction: predictors and long-term mortality. Int J Cardiol Heart Vasc.

[15] Schömig A, Mehilli J, Antoniucci D (2005). Mechanical reperfusion in patients with acute myocardial infarction presenting more than 12 hours from symptom onset: a randomized controlled trial. JAMA.

[16] Gibson CM, de Lemos JA, Murphy SA (2001). Combination therapy with abciximab reduces angiographically evident thrombus in acute myocardial infarction: a TIMI 14 substudy. Circulation.

[17] World Medical Association (2013). World Medical Association Declaration of Helsinki: ethical principles for medical research involving human subjects. JAMA.

[18] van't Hof AWJ, Liem A, Suryapranata H, Hoorntje JCA, de Boer. MJ, Zijlstra F (1998). Angiographic assessment of myocardial reperfusion in patients treated with primary angioplasty for acute myocardial infarction: myocardial blush grade.

[19] Lawton JS, Tamis-Holland JE, Bangalore S (2022). 2021 ACC/AHA/SCAI guideline for coronary artery revascularization: a report of the American College of Cardiology/American Heart Association joint committee on clinical practice guidelines. Circulation.

[20] Qiao J, Pan L, Zhang B (2017). Deferred versus immediate stenting in patients with ST-segment elevation myocardial infarction: a systematic review and meta-analysis. J Am Heart Assoc.

[21] Lee JM, Rhee TM, Chang H (2017). Deferred versus conventional stent implantation in patients with acute ST-segment elevation myocardial infarction: an updated meta-analysis of 10 studies. Int J Cardiol.

[22] Magdy AM, Demitry SR, Hasan-Ali H, Zaky M, Abd El-Hady M, Abdel Ghany M (2021). Stenting deferral in primary percutaneous coronary intervention: exploring benefits and suitable interval in heavy thrombus burden. Egypt Heart J.

[23] Ghazal KHM, Hussein EM, Mohammad MG, Hozien WR (2024). Outcome of immediate versus delayed stenting in ST-segment elevation myocardial infarction patients with high thrombus burden. Zagazig Univ Med J.

[24] Liu R, Xu F, Liu T, Zhou Y, Wu X (2024). Efficacy and safety of deferred stenting in geriatric patients with STEMI and high thrombus burden. Rev Cardiovasc Med.

[25] Tang L, Zhou SH, Hu XQ, Fang ZF, Shen XQ (2011). Effect of delayed vs immediate stent implantation on myocardial perfusion and cardiac function in patients with ST-segment elevation myocardial infarction undergoing primary percutaneous intervention with thrombus aspiration. Can J Cardiol.

[26] Jolicoeur EM, Dendukuri N, Belisle P (2020). Immediate vs delayed stenting in ST-elevation myocardial infarction: rationale and design of the international PRIMACY Bayesian randomized controlled trial. Can J Cardiol.

